# Advancements on Single-Atom Catalysts-Mediated Persulfate Activation: Generating Reactive Species for Contaminants Elimination in Water

**DOI:** 10.3390/molecules29235696

**Published:** 2024-12-02

**Authors:** Wan Yu, Yin Xu

**Affiliations:** Hubei Key Laboratory of Regional Development and Environmental Response, Faculty of Resources and Environmental Science, Hubei University, Wuhan 430062, China; 15327147626@163.com

**Keywords:** single-atom catalyst, persulfate, reactive species, influencing factors, pollutants

## Abstract

The single-atom catalyst (SAC) activated persulfate process has emerged as a highly efficient technology for eliminating refractory organic compounds in aqueous environments. This review delves into the intricacies of utilizing SACs for the effective removal of various contaminants in water. The common supports and the preparation procedures of SACs are summarized at first. The synthesis methods of SACs (i.e., wet chemical method, one-pot hydrothermal method, and high-temperature pyrolysis method) are also described. Then, a comprehensive overview of the diverse reaction mechanisms in SAC-activated persulfate systems is presented, including a radical oxidation process via sulfate or hydroxyl radicals and superoxide radicals, or a nonradical process via single oxygen, surface active complex, and high-valent metal-oxo species oxidation. The impact of key factors such as peroxides concentration, SAC dosage, reaction pH, inorganic anions, organic matter, operando stability, and real water is also delved. The removal of various pollutants (i.e., azo dyes, phenolic compounds, pharmaceuticals, and bacteria) by this process is further summarized. Finally, the challenges and perspectives in the field of water treatment utilizing SACs are discussed.

## 1. Introduction

Currently, there exist vast amounts of organic pollutants that are hard to break down, such as phenolic compounds, organochlorine pesticides, and pharmaceuticals, which are persistent, toxic, and carcinogenic, posing extreme harm to people, animals, and plants [1,2]. Conventional treatment methods including coagulation-flocculation, precipitation, adsorption, and membrane separation are widely used in sewage treatment. However, these methods require a large consumption of chemical reagents, energy, as well as the regular replacement or maintenance of equipment, resulting in high costs [3,4,5,6,7]. More important is that conventional treatments are unable to completely eliminate these types of organic pollutants from water [8]. Therefore, a technology which is low-cost and recyclable and can effectively remove pollutants needs to be developed.

Researchers have demonstrated that persulfate-mediated advanced oxidation processes (PS-AOPs) have the capability to oxidize organic contaminants that are challenging to be degraded in complex water systems. These processes generate reactive oxygen species (ROS) to break down organic pollutants via the activation of persulfate, specifically peroxymonosulfate (PMS) and peroxydisulfate (PDS), by catalysts [9,10]. PMS (H-O-O-SO_3_^−^) has an asymmetric structure with a longer O-O bond (1.326 Å), while PDS (SO_3_^−^-O-O-SO_3_^−^) possesses a symmetric structure with a shorter O-O bond length of 1.322 Å, which probably implies that PMS is more reactive and more easily activated by catalysts than PDS [11]. Typically, the catalysts used in these reactions contain homogeneous metal ions, heterogeneous solids such as zero-valent metals (i.e., Fe nanoparticles [12]), metal oxides (i.e., ZnO [13], MnO_2_ [14]), and metal oxide composites (i.e., Ca_2_Co_2_O_5_ [15], Co_3_O_4_/MnO_2_ [16]) and so on. The heterogeneous catalysis system has been considered to be a more promising method because the solid catalyst is easy to be separated from the reaction medium and can be recycled, which helps reduce secondary pollution caused by residual catalyst in solution [17,18]. However, heterogeneous catalysis is essentially a surface process and its effectiveness is limited by surface accessibility. Taking loaded metal nanoparticles as an example, the size of the metallic nanostructures plays an important role in catalytic performance. For the use of metal-nitrogen-carbon (M-N-C) catalysts via PMS activation to degrade contaminants, the order of catalytic activity over metal sites in M-N-C catalysts is believed to be single atoms (~0.1 nm) > atomic clusters (~1 nm) > nanoparticles (over 5 nm) [19]. By reducing the particle size of the catalyst, the surface coordinatively unsaturated metal atoms, which function as the primary catalytic sites, can significantly enhance the intrinsic activity per metal atom [20].

Recent research has reduced anchored metal nanoparticles to the atomic level, resulting in the production of isolated single-atom metal sites without metal-metal bonds over support material, which are referred as single-atom catalysts (SACs) [21,22,23]. SACs combine the high atom utilization of homogeneous catalysts with the ease of recovery associated with heterogeneous catalysts [24]. They have demonstrated widespread application in diverse catalytic processes, particularly in the oxygen reduction reaction [25], hydrogen evolution reaction [26], CO_2_ reduction reaction [27], and organic synthesis [28]. Furthermore, SACs have been investigated for their potential in pollutant removal technologies based on advanced oxidation processes (AOPs). SACs offer several advantages over common nanocatalysts: (1) they exhibit faster reaction rates due to higher atom utilization; (2) most SACs are more cost-effective to prepare owing to requiring less material; (3) SAC-activated systems possess strong selectivity due to the mononuclear nature of the active sites, which probably leads to selectivity in reactive species generation [29], attacking targeted pollutants in complex water matrices; and (4) the surface active sites demonstrate greater stability due to the strong covalent bonding between the individual metal atom and the support, which results in minimal leaching of metal ions during the reaction process, thereby reducing the occurrence of secondary environmental contamination. These unique advantages have led to SAC-based AOPs rapidly developing in recent years. Some relevant review articles are summarized in Table 1. As can be seen, these studies primarily concentrated on exploring the synthetic methodologies and characterization techniques of SACs, along with their application in peroxides activation, photocatalysis, and electrocatalysis for wastewater treatment. Additionally, the associated catalytic mechanisms underlying these processes were also investigated [30,31,32,33,34,35,36]. 

However, a comprehensive summary overview of the sole persulfate activation by SACs in the absence of any external energy remains scarce in the existing literature. There remains a significant knowledge gap in understanding the mechanisms and influencing factors of SAC-activated persulfate.

In this paper, we present a summary of the latest developments in the removal of environmental pollutants utilizing SAC-activated PMS and PDS technologies. The review will outline the various supports and synthesis processes of SACs, review the main active species in SAC-activated persulfate systems, analyze the impacts of reaction parameters, and provide an overview of the application of this technology in pollutant treatment. 

## 2. Supports and Synthesis Methods of SACs

Recent studies have reported a variety of supports to prepare SACs for persulfate-activation, such as metal (hydr)oxides [37,38]/sulfides [39,40]/nitrides [41,42] and carbon matrix materials [43,44]. The structure, surface area, and characteristics of common support materials are provided in Table 2. The above-mentioned supports always create anchor sites by bringing in electronic/structural disorder, which is the most common strategy for obtaining SAC materials. The interaction between the support and a single atom is strong enough to hinder the formation of clusters, yet weak enough to prevent serious disturbances for the electronic configuration of atoms, thereby retaining the catalytic activity. Additionally, supports provide a large specific surface area for the active substance and control the geometric and electronic structure of SACs. Therefore, selecting the most appropriate support material is a crucial step in the preparation of SACs [45]. 

### 2.1. Metal Hydroxide/Sulfide/Nitride-Supported SACs

Metal hydroxides, sulfides, and nitrides have been widely used as supports for SACs. They offer significant advantages in stabilizing single metal atoms over alternative supports, owing to their large specific surface areas, plentiful metal or non-metal vacancies, and rich surface functional groups [35]. 

Layered double hydroxides (LDHs), two-dimensional (2D) metal hydroxides, have been employed to anchor single atoms. These LDHs possess defect sites and hydroxyl groups (-OH) on their surfaces which can form coordination bonds with single-atom metal atoms to bond single-atomic metal atoms, lattice oxygen, and metal ions of LDHs [53,54]. Tan et al. prepared atomically dispersed Ru on NiFe-LDH (Ru-LDH) using a wet chemical method. The NiFe-LDH precursor was prepared by mixing Fe(NO_3_)_3_·9H_2_O, Ni(NO_3_)_2_·6H_2_O, NaOH, and Na_2_CO_3_ at a temperature of 65 °C for 18 h. The NiFe-LDH was subsequently immersed in a solution containing NaOH and RuCl_3_·3H_2_O at a temperature of 50 °C for a duration of 8 h. This process resulted in the synthesis of Ru-LDH with a concentration of 0.77 wt% Ru [37]. Chen et al. prepared Co-MgAl-LDH by subjecting it to calcination at 550 °C in a N_2_ environment. The purpose of this process was to enhance the bonding between single Co atom and LDH [38]. Nevertheless, a high temperature deoxygenation process may potentially lead to the destabilization of LDH-based SACs, as metal ions within the LDH structure are also likely to be reduced to neutral metals, resulting in the formation of undesired single-atom impurities [35]. 

**Figure 1:**
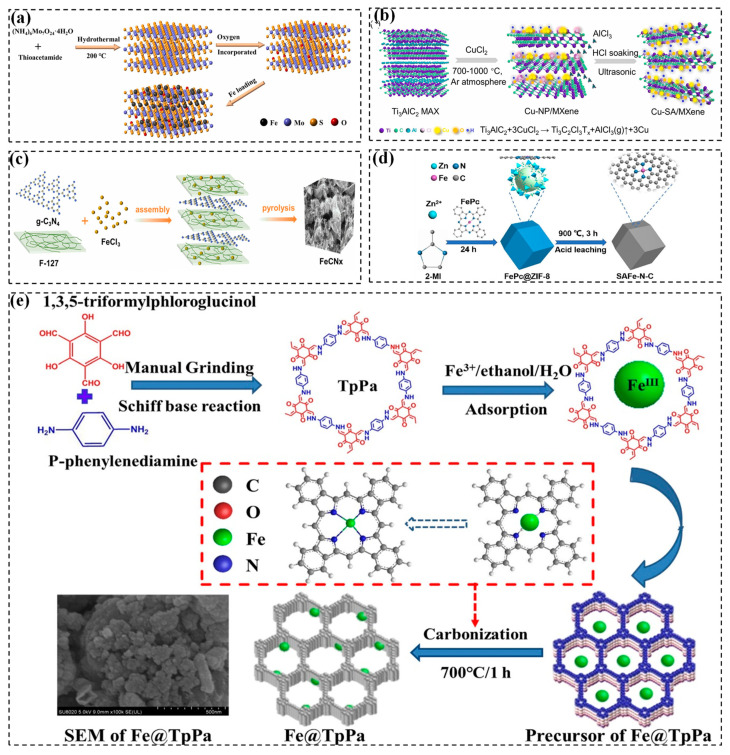
(**a**) Schematic diagram for the synthesis of Fe-MoS_2_ [39]. Copyright 2023 Elsevier. (**b**) Schematic illustration for the synthesis of Cu-SA/MXene catalysts [42]. Copyright 2023 Elsevier. (**c**) Schematic of FeCNx synthesis [64]. Copyright 2021 Elsevier. (**d**) Schematic diagram of SA Fe-N-C preparation process [71]. Copyright 2021 Elsevier. (**e**) Illustrative procedure for the fabrication of Fe@COF catalysts [77]. Copyright 2019 Elsevier.

Apart from metal hydroxides, the metal sulfide MoS_2_ can also be employed as an excellent 2D supporter to synthesize metal SACs. MoS_2_ is composed of two layers of sulfur atoms sandwiching a middle layer of molybdenum atoms, held together by Van Der Waals forces. Recent years have seen the anchoring of Fe and Co atoms in MoS_2_ to synthesize SACs. For instance, Huang et al. demonstrated the synthesis of single Fe atoms confined within two-dimensional MoS_2_ nanosheets (Fe_x_Mo_1–x_S_2_) using a one-pot hydrothermal method. In this work, FeSO_4_, Na_2_MoO_4_, and L-cysteine were used as the sources of Fe, Mo, and S, respectively. L-cysteine undergoes thermal breakdown, resulting in the production of hydrogen sulfide (H_2_S), which subsequently reduces MoO_4_^2−^ to MoS_2_. The free sulfur termini of MoS_2_ is also assumed to be collided with Fe^2+^, facilitating the introduction of Fe atoms into MoS_2_ nanosheets [55]. Additionally, Chen et al. also created xFe-MoS_2_ nanoflowers by incorporating individual Fe atoms into MoS_2_ nanosheets. Specifically, MoS_2_ were initially synthesized by a hydrothermal of (NH_4_)_6_Mo_7_O_24_·4H_2_O and thioacetamide, followed by partial oxidation of MoS_2_ to MoO_2_. Then, Fe^2+^ was reduced to individual Fe atoms by potassium borohydride (KBH_4_) on the surface of MoS_2_ particles, resulting in a uniform distribution of Fe across the MoS_2_ surface (Figure 1a) [39]. Two-dimensional MoS_2_ sheets with abundant confinement sites facilitate atomic uniform dispersion, which significantly improves atom utilization. 

Another two-dimensional carrier, MXenes (MAX), represent a recent model ternary transition metal carbide and nitride, in which M, A and X denote early transition metals, IIIA or IVA elements, and C or N, respectively [41,56]. MXenes, such as Ti_2_AlN and Ti_3_AlC_2_, have been proved as monoatomic catalyst carriers due to their capacity to stabilize a single metal atom through strong metal-carrier interaction, high electrical conductivity, superior corrosion resistance, and abundant surface metal active sites [35]. The synthesis process of atomic copper anchored on Ti_3_AlC_2_ MXene nanosheets is shown in Figure 1b. Molten chloride salts, specifically CuCl_2_, etch Al layers and a few Ti atoms of Ti_3_AlC_2_ to generate Ti vacancy defects under Ar gas at temperatures of 700–1000 °C. This is followed by soaking in an HCl solution to remove Cu particles, resulting in the formation of Cu-SA/MXene. After treatment with a high pyrolysis temperature and etching process, the density of defects on the Ti_3_AlC_2_-MXene surface increases, which favors the stability of isolated Cu atoms [42]. Similarly, molten CoCl_2_ salt was employed to etch Al layers of Ti_2_AlN to obtain the single-atom catalyst Co@Ti_2–x_N [41]. Currently, TiAlX is frequently employed as a support of metal single atoms. It is crucial to further investigate the potential use of other MXene materials to anchor single atoms. 

### 2.2. Carbon-Supported SACs

Carbon-supported SACs typically comprise carbon frameworks and metal dopants. The presence of strong metal-carrier interactions between individual metal atoms and the carbon substrate serves two purposes: preventing the clustering of metal atoms and modulating geometric structures and electronic configurations of the catalytic sites. Carbon nanomaterials, unlike metal hydroxides, exhibit strong covalent bonding between carbon atoms. This unique characteristic endows them with an exceptional electrical conductivity, which enhances electron transport between reactants and active sites, ultimately resulting in good catalytic performance in various energy conversion reactions [57,58]. Furthermore, carbon-supported SACs demonstrate exceptional catalytic activity in biomedical applications due to their outstanding biodegradability and biocompatibility [59]. The synthesis processes of SACs supported by different carbon materials, such as g-C_3_N_4_, COF, MOF, and biomass, are summarized below. 

#### 2.2.1. g-C_3_N_4_

Graphitic carbon nitride (g-C_3_N_4_), a novel category of nitrogen-doped carbon materials with a two- or three-dimensional structure, is composed of lamellar hexagonal building blocks of triazine units, forming a lamellar π-conjugated structure consisting of sp^2^C and sp^2^N [60,61]. In contrast to common nitrogen-doped carbons with disorganized structures and low N species content, g-C_3_N_4_ possesses well-organized pore structures and a higher number of N atoms as active sites. Hence, g-C_3_N_4_ is extensively employed as a medium for attaching isolated metal atoms. The supramolecular gel-assisted thermal polymerization method is a common approach for synthesizing single-atom catalysts anchored on g-C_3_N_4_. g-C_3_N_4_ is typically synthesized by the high temperature calcination of melamine or urea, and then metal salt ions and organometallic compounds are often applied as sources of single metal atoms [62]. Besides, a water-soluble surfactant F127 (polyoxyethylene-polyoxypropylene-polyoxyethylene) is reported to disperse iron atom for facilitating the formation of Fe-Nx moieties [63]. Specifically, g-C_3_N_4_ is prepared using a conventional polycondensation of urea at 550 °C for 2 h. This is then mixed with F127 and FeCl_3_ and the mixture subsequently carbonized at 600 °C in an Ar atmosphere to obtain the FeCN_x_ catalysts (Figure 1c) [64]. During the synthesis process, the plentiful and periodically spaced N atoms in g-C_3_N_4_ play a pivotal role in stabilizing the single atoms by utilizing the electron lone pairs of N as anchor sites.

#### 2.2.2. MOF

Metal organic frameworks (MOFs) are metal-organic skeletons formed through self-assembly, with metal ions or clusters serving as nodes and organic ligands acting as connecting units that held together by coordination bonds. Owing to their structural advantages, including flexible nanostructures, high specific surface area, and high porosity, MOFs are considered suitable precursors for SACs synthesis [43]. MOF-based SACs have found extensive applications in electro/photocatalysis [65,66], batteries [67], and nanomedicine [68]. MOFs can spatially isolate and encapsulate suitable mononuclear metal precursors because of the pore confinement effect. To spatially uniform disperse metal precursors, it is essential that the diameter of the metal precursor is slightly smaller than the pore diameter of the MOFs, allowing the metal precursor to be trapped within the pores and preventing easy release. Additionally, MOFs can be calcined to remove the ligands, generating numerous coordination sites that serve as anchor sites for metal atoms. For instance, subjecting MOFs that contain N ligands to high-temperature pyrolysis can result in the formation of nitrogen-doped porous carbon materials. The presence of a large amount of nitrogen species can serve as an anchor for individual atoms to stabilize SACs [69].

ZIF-8, a zinc-based zeolitic imidazole framework, exhibits the ability to trap single-metal precursors inside its cavity by the microporous confinement effect [70]. The synthetic pathway involved in situ encapsulation is followed by high temperature pyrolysis. Initially, ZIF-8 was synthesized by the chemical reaction of Zn(NO_3_)_2_·6H_2_O and 2-methylimidazole. Then, ZIF-8 was added into a solution containing various transition metal sources, including iron phthalocyanine (FePc) [44,71], Fe(NO_3_)_3_ [72], Co(acac)_2_ [73], and Ru(acac)_3_ [74]. These precursors were then annealed in an inert gas environment to remove the ligand and generate isolated single atoms. As depicted in Figure 1d, FePc encapsulated in ZIF-8 was reduced to individual Fe atoms during the carbonation of ZIF-8 organo-conjugate. The Fe atoms were anchored by the abundant N species to yield a monoatomic catalyst (SAFe-N-C) [71]. Notably, the Zn atoms in ZIF-8 with a low boiling point could be selectively evaporated during pyrolysis to create defects and vacancies. This process not only generated more free N sites, but also increased the distance of adjacent Co atoms, therefore avoiding the formation of Co-Co bonds at high temperatures [32]. Nevertheless, this synthesis technique necessitates the initial synthesis of both the MOF precursor and polymer, followed by a heat treatment process, making it economically unviable. Therefore, improved techniques such as acid treatment deserve to be investigated, as they can efficiently remove excess metal nanoparticles (NPs) and leave well-dispersed metal atoms on MOF-supported catalysts [75]. 

#### 2.2.3. COF

Covalent organic frameworks (COFs) are topologically connected organic structures with strong covalent bonds [76]. Similar to MOFs, COFs also have a distinct porosity structure that allows for the spatial confinement of mononuclear metal precursors. Of note, COFs incorporate various light elements, including boron (B), carbon (C), nitrogen (N), and oxygen (O), which can serve as coordination sites to stabilize individual metal atoms, preventing their migration and agglomeration [69]. Yao et al. successfully confined single-atom Fe within a porous carbon matrix (denoted as Fe@TpPa), forming an efficient catalyst for the oxidation of aqueous contaminants (Figure 1e). As can be seen, P-phenylenediamine (Pa) and 1,3,5-trifoyl phloroglucinol (Tp) are ground into fine powder, followed by the dropwise addition of acetic acid as a catalyst in a solvent containing mesitylene and dioxane, resulting in a dark red powder known as TpPa. Subsequently, TpPa powder and FeCl_3_ was mixed in a methanol solution to obtain Fe@COF, followed by calcination in a N_2_ atmosphere at 700 °C to form a monatomic iron catalyst. The total Fe content was ca. 2.14 wt%, with Fe atoms dispersed in an Fe-Nx configuration [77]. Nevertheless, while COF-based SACs exhibit high metal loading and exceptional atomic dispersion, a fraction of the metal atoms remains inactive. Hence, it is imperative to design COF substrates with higher surface densities to effectively isolate and optimize the metal loading for enhanced catalytic performance. 

#### 2.2.4. Biomass-Based Materials

Biomass feedstocks are being harnessed as substitutes for fossil energy sources in the manufacture of fuels, chemicals, and materials to efficiently mitigate energy demands in response to carbon neutrality and peak carbon regulations [78]. Recently, there has been a growing interest in the biomass preparation for SACs. Functional groups presenting in biomass, such as -OH, -COOH, -NH_2_, have the ability to fix metal ions and prevent the aggregation of carbon carriers. To date, spirulina, lignin, and chitosan have been used as biomass feedstock precursors for preparing SACs [79,80,81]. For instance, lignin, with its 3D amorphous structure rich in diverse functional groups, can easily form insoluble supramolecular metal-lignin complexes through coordination with transition metal ions. Thus, lignin is regarded as a “spider web” for capturing atomic metals such as Fe and Co [81,82,83]. Interestingly, the organic iron presenting in *Enteromorpha* has been used as a metal source for forming carbonous Fe-SACs, which is conducive to sustainable and green development by utilizing the intrinsic metal elements within biomass, rather than introducing external metal salts [84]. Specifically, *Enteromorpha* was pyrolyzed at 900 °C for 2 h in a N_2_ atmosphere and then soaked in a H_2_SO_4_ solution overnight to get rid of unstable metal species. Ultimately, an iron loading of 0.84 wt% was achieved by anchoring single Fe sites to N-doped carbon substrates. However, the widespread use of biomass-supported SACs is hindered by the low content of intrinsic metal elements in biomass compared to other supports that can achieve high loadings (>15 wt%) [32]. So far, only a small amount of biomasses, such as lignin and chitosan, has been conveniently and stably converted into SACs in the laboratory with low loadings (<5 wt%). Therefore, there is an urgent need to investigate new technologies to address this challenge.

## 3. Dominant Reactive Species Generation

The activation of persulfate by SACs has been assumed to generate a diverse range of ROS, which are highly effective in removing refractory organic contaminants from water via both radical and nonradical oxidation mechanisms. SACs used in the elimination of contaminants with different reactive species generation have been summarized in Table 3. The primary radicals involved are hydroxyl radicals (^•^OH), sulfate radicals (SO_4_^•−^), and superoxide radicals (O_2_^•−^), while nonradical pathways encompass singlet oxygen (^1^O_2_), electron transfer, and high-valent metal-oxo species. Radical oxidation exhibits a superior capacity for mineralizing organic pollutants. Meanwhile, nonradical oxidation offers advantages such as avoiding the self-quenching of radicals and reducing interference from the complex compounds present in water [9]. Therefore, the simultaneous coexistence of radical and nonradical pathways in the oxidation process provides certain benefits for refractory pollutants elimination. 

### 3.1. Radicals

**Figure 2:**
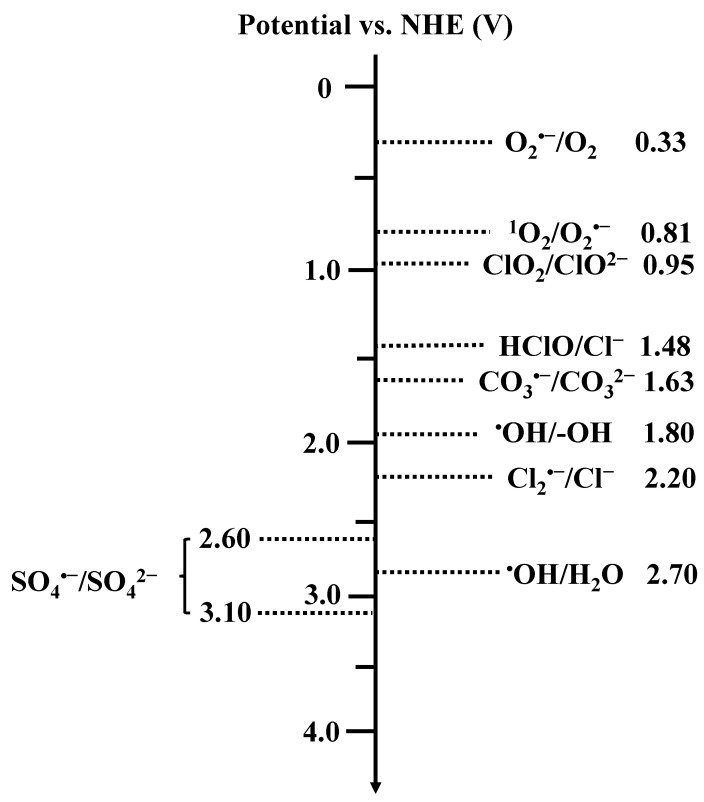
The band diagram energy of common reactive oxidation species.

Catalyst-mediated persulfate activation is a popular strategy to produce radicals (i.e., SO_4_^•−^, ^•^OH, and O_2_^•−^) for highly efficient pollutant degradation. As shown in Figure 2, the stronger oxidizing potential and longer half-life of SO_4_^•−^ (2.60~3.10 V vs NHE, 30~40 μs) compared to ^•^OH (1.80~2.70 V vs. NHE, 20 ns) endow it with the capability to degrade target pollutants over longer distances and mineralize them more effectively than ^•^OH [32,109]. In many cases, SO_4_^•−^, ^•^OH, and O_2_^•−^ were generated simultaneously to synergistically oxidize pollutants.

To validate the radicals’ generation, the quenching agent are introduced into the system, inhibiting the degradation efficiency of pollutants. Radical scavengers, such as p-benzoquinone (p-BQ, $k_{O_{2}^{•-}}$_/p-BQ_ = 1.0 × 10^9^ M^−1^s^−1^), methanol (MeOH, $k_{{SO}_{4}^{•-}}$_/MeOH_ = 3.2 × 10^6^ M^−1^s^−1^, $k^{•}$_OH/MeOH_ = 9.7 × 10^8^ M^−1^s^−1^) and isopropyl alcohol (IPA, $k_{{SO}_{4}^{•-}}$_/IPA_ = 8.5 × 10^7^ M^−1^s^−1^, $k^{•}$_OH/IPA_ = 1.9 × 10^9^ M^−1^s^−1^), are always utilized to quench O_2_^•−^ and both SO_4_^•−^/^•^OH, respectively. In contrast, tert-butanol (TBA, $k^{•}$_OH/TBA_ = (3.8–7.6) × 10^9^ M^−1^s^−1^) selectively quenches ^•^OH [30,110]. Quantifying the relative contribution efficiency of free radicals is crucial for understanding the degradation mechanism, which can be deduced using Equations (1)–(3) [111]. It should be noted that Equation (1) will be invalid when TBA exhibits a higher inhibition effect than MeOH, likely due to TBA’s stronger hydrophobicity causing its enhanced adsorption on the surface of catalyst, thus hindering the interaction of oxidants/pollutants with catalysts [71].

(1) $$R_{{SO}_{4}^{•-}}=\frac{k_{TBA}-k_{MeOH}}{k}$$

(2) $${R^{•}}_{\mathrm{OH}}=\frac{{k^{•}}_{\mathrm{OH}}}{k}=\frac{k-k_{\mathrm{TBA}}}{k}$$

(3) $$R_{O_{2}^{•-}}=\frac{k_{O_{2}^{•-}}}{k}=\frac{k-k_{p-\mathrm{BQ}}}{k}$$

Electron paramagnetic resonance (EPR) analysis is a visualization approach for confirming the existence of radicals. The EPR spectrum displays the characteristic signals of 5,5-dimethyl-1-pyrroline-N-oxide (DMPO) adducts formed with SO_4_^•−^, ^•^OH, and O_2_^•−^ [82]. The enhanced signals of DMPO-SO_4_^•−^, DMPO-^•^OH, and DMPO-O_2_^•−^ in the presence of both SACs and PMS compared to PMS alone indicate the formation of these radicals during the reaction [85]. The addition of radical scavengers leads to a reduction in these signals, confirming their roles as primary active species [86]. However, DMPO-SO_4_^•−^ adducts tend to rapidly transform to DMPO-^•^OH through a nucleophilic substitution reaction, making it challenging to detect DMPO-SO_4_^•−^ signals [87,112]. Conversely, radical probe compounds such as atrazine (ATZ, probe for SO_4_^•−^ and ^•^OH) and nitrobenzene (NB, probe for ^•^OH) were employed to accurately identify the presence of radicals [41]. 

**Figure 3:**
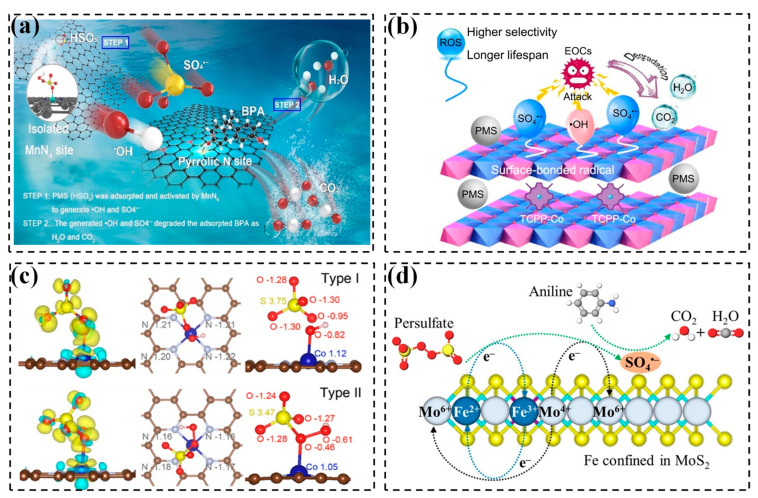
(**a**) The proposed mechanism of BPA removal in the Mn-ISAs@CN/PMS systems [88]. Copyright 2020 Elsevier. (**b**) Removal mechanism of EOCs in SA-Co-LDH/PMS system [38]. Copyright 2024 Elsevier. (**c**) Adsorption configuration and charge density of PMS on catalyst surface with H-adjacent (Type I) and S-adjacent (Type II) O atom in peroxide bond [86]. Copyright 2021 American Chemical Society. (**d**) Degradation mechanism of aniline in FexMo_1–x_S_2_/PDS system [40]. Copyright 2020 Elsevier.

The generation of radicals in SAC-activated PS systems typically involves two steps: (i) enhanced adsorption of PS on metal atoms via electrostatic attraction and (ii) accelerated O-O bond cleavage in PS via electron transfer [86]. PMS can be directly absorbed on the metal active sites, altering the local electron distribution and remarkably stretching the O-O bond, facilitating its cleavage to generate ^•^OH and SO_4_^•−^ (Equations (4)–(6)) [41,113,114]. Yang et al. have reported the adsorption and activation of PMS by a Mn-based SAC, generating ^•^OH and SO_4_^•−^ that subsequently degraded BPA to H_2_O and CO_2_ (Figure 3a) [88]. Further, Chen et al. found the in situ generation of surface-bonded ^•^OH and SO_4_^•−^ via PMS activation by SA-Co sites confined in LDHs (Figure 3b). These kinds of radicals have the ability to persist for an extended period of time (up to 48 h) due to the strong attraction between the radicals and LDHs, which suppresses the radicals’ self-quenching and enhances their interaction with pollutants, thereby enhancing the utilization efficiency of oxidative species [38]. Chu et al. further tried to reveal the generation mechanism of ^•^OH and SO_4_^•−^, respectively, based on the type of oxygen atom in the peroxide bond absorbed by the metal sites. Specifically, two types of adsorption were identified: Type I, involving the O atom adjacent to the H atom, and Type II, involving the O atom adjacent to the S atom (Figure 3c). In Type I adsorption, PMS acquires electrons from π-conjugation within the support, enabling the cleavage of the O-O bond (2.01 Å) of PMS and then breaking down into surface-bonded ^•^OH and free SO_4_^•−^. Conversely, in Type II adsorption, the surface-bonded SO_4_^•−^ and free ^•^OH are assumed to be main reactive species. In this case, the charge transfer is minimal and the O-O bond of PMS undergoes a slight elongation from 1.44 to 1.46 Å, which is not sufficient for the direct dissociation of the O-O bond. Hence, the absorption type between the metal site and O-O bond is crucial for the formation of surface radicals in SAC-activated persulfate systems [86]. However, in the case of PDS, both O atoms in the O-O bond exhibit equal absorption capacity due to its symmetrical structure, suggesting that a uniform outcome will be obtained regardless of the oxygen atom involved in the absorption process. Notably, the formation of SO_4_^•−^ always results from the transfer of electrons from SACs to PDS, as described in Equation (7) [89].

For the generation of O_2_^•−^, the most common reaction is the reduction of dissolved oxygen (DO) by oxygen vacancies on the catalyst surface or electron-rich metal centers Equation (8) [77,111]. In addition, ^•^OH also plays a vital role in the production of O_2_^•−^ by reactions (9) and (10). PDS can even donate electrons to catalysts for O_2_^•−^ generation (Equations (11)–(12)) [89,115].

M^n+^ + HSO_5_^−^ → M^(n+1)+^ + SO_4_^•−^+ OH^−^ (4)

M^n+^ + HSO_5_^−^ → M^(n+1)+^ + ^•^OH + SO_4_^2−^ (5)

SO_4_^•−^ + H_2_O → SO_4_^2−^ + ^•^OH + H^+^ (6)

S_2_O_8_^2−^ + e^−^ → SO_4_^2−^ + SO_4_^•−^ (7)

O_2_ + e^−^ → O_2_^•−^ (8)

HSO_5_^−^+ ^•^OH → SO_4_^2−^ + HO_2_^•^ + H^+^ (9)

HO_2_^•^ → O_2_^•−^ + H^+^ (10)

S_2_O_8_^2−^ + 2H_2_O − e^−^ → 2SO_4_^2−^ + O_2_^•−^ + 4H^+^ (11)

S_2_O_8_^2−^ + Fe(III)-g-C_3_N_4_ + H_2_O → Fe(II)-g-C_3_N_4_ + 2SO_4_^2−^ + 1/2O_2_^•−^ + 4H^+^ (12)

The synergistic effect over dual metal sites has been found to further enhance catalytic activity of SACs [45]. For example, the dual Fe and Mo sites in Fe_x_Mo_1-x_S_2_ can efficiently activate PDS to degrade aniline (Figure 3d), with >Fe(II) and >Mo(IV) initially being reacted by S_2_O_8_^2−^ to >Fe(III) and >Mo(VI) to generate SO_4_^•−^. Subsequently, Fe(III) can be reduced into >Fe(II) by >Mo(VI) to continuously activate PDS in producing SO_4_^•−^, which serves as the primary active species (Equations (13)–(15)) [40]. The interaction between Fe and Mo significantly improves the activation efficiency of PDS.

>Fe(II) + S_2_O_8_^2−^ → >Fe(III) + SO_4_^2−^ + SO_4_^•−^ (13)

>Mo(IV) + S_2_O_8_^2−^ → >Mo(VI) + SO_4_^2−^ + SO_4_^•−^ (14)

>Fe(III) + >Mo(IV) → >Fe(II) + >Mo(IV)(15)

### 3.2. Singlet Oxygen

Single oxygen (^1^O_2_) has emerged as a high-efficiency reactive species for the oxidation of emerging contaminants. Despite ^1^O_2_ possessing lower oxidizing potential (2.2 V vs. NHE) compared to SO_4_^•−^ and ^•^OH [71], it exhibits exceptional selectivity in oxidizing unsaturated organic compounds through electrophilic addition and electron extraction mechanisms [11], making it a primary reactive species in SAC-based persulfate activation processes. 

Chemical scavengers including NaN_3_ (kO12_/NaN_3__ =1 × 10^9^ M^−1^s^−1^), furfuryl alcohol (FFA, kO12_/FFA_ = 1.2 × 10^8^ M^−1^s^−1^), and L-histidine (kO12_/L-histidine_ = 3.2 × 10^7^ M^−1^s^−1^) have been utilized to verify the existence of ^1^O_2_ in SAC-activated persulfate systems [90,91,116]. Liao et al. reported that the presence of FFA inhibited acetaminophen removal. In addition, the removal efficiency of the pollutant significantly decreased with increasing FFA concentration, suggesting that ^1^O_2_ dominated in the degradation of acetaminophen [92]. However, it is crucial to note that L-histidine may consume PMS, thereby inhibiting degradation efficiency. Additionally, FFA may compete with persulfate molecules for adsorption on the surface active site of catalyst instead of direct reaction with ^1^O_2_, leading to potentially inaccurate results in scavenging experiments [90]. 

To further substantiate the presence of ^1^O_2_, 2,2,6,6-tetramethylpiperidine (TEMP) is utilized as a trapping agent to detect ^1^O_2_ for EPR analysis. The triplet signals with an intensity ratio of 1:1:1 imply ^1^O_2_ production [92]. Descending signals of TEMP in the presence of the target pollutant further confirms the participation of ^1^O_2_ towards contaminant degradation. However, some reactive species may directly oxidize TEMP via deprotonation and O_2_ addition, finally forming the same EPR signal of ^1^O_2_ [82]. Therefore, a combination of scavenging experiments and EPR analysis is recommended to conclusively prove ^1^O_2_ generation.

**Figure 4:**
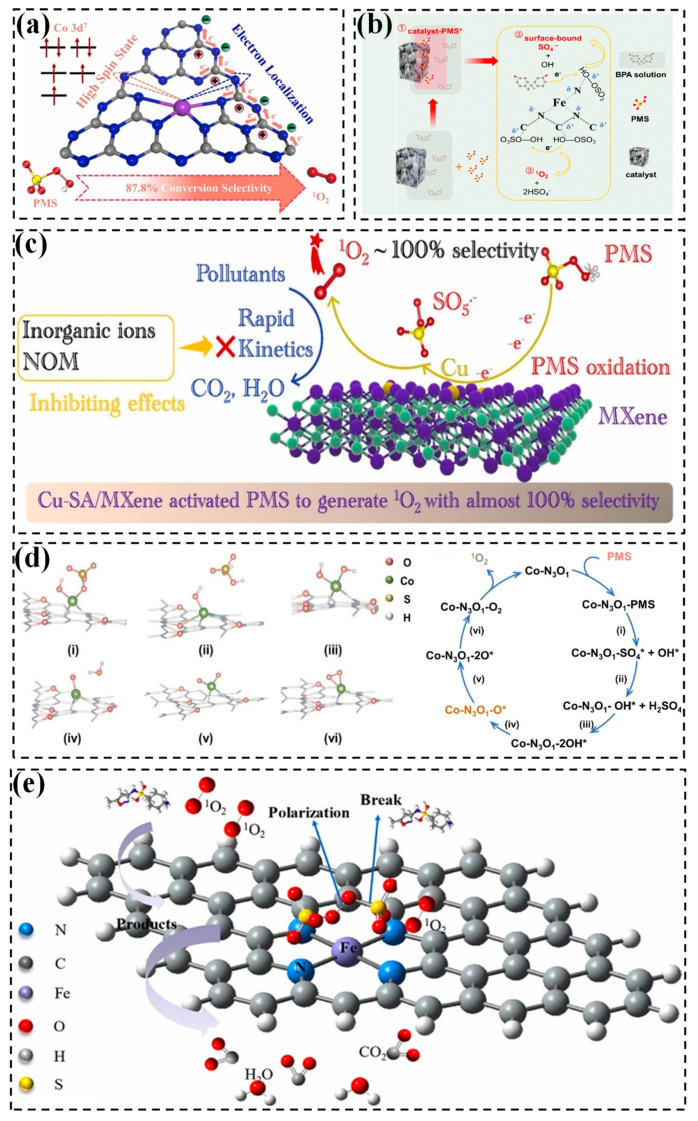
(**a**) Removal mechanism of 17β-estradiol in the Co-SA/CMN/PMS system [118]. Copyright 2023 Elsevier. (**b**) Nonradical mechanism of PMS activation by Fe-N-doped carbon catalyst [64]. Copyright 2021 Elsevier. (**c**) Mechanism of PMS activation by Cu-SA/MXene catalyst [42]. Copyright 2023 Elsevier. (**d**) The proposed reaction mechanism in the Co-N_3_O_1_/PMS system [94]. Copyright 2022 Wiley-VCH. (**e**) The mechanism of PDS activation by Fe(MIL)-SAC catalyst [97]. Copyright 2023 Elsevier.

The generation of ^1^O_2_ is facilitated by a strong metal-O (where O originates from PMS) bonding strength, which avoids spontaneous dissociation of adsorbed PMS molecules into ^•^OH and SO_4_^•−^ [117]. Wang and coworkers have developed single Co atoms anchored on an uneven g-C_3_N_4_ nanosheet and found that 87.8% PMS was consumed for the selective generation of ^1^O_2_ to degrade multiple organic pollutants. The possible explanation is that the distribution of positive and negative charge centers on the carriers can avoid the spatial site resistance and electrostatic repulsion of the PMS molecules. Furthermore, the high spin state of the metal atoms improves the electron transfer in the spin orientation, enhancing the chemisorption and dissociation of PMS on Co sites (Figure 4a) [118]. 

Several routes for ^1^O_2_ production during persulfate activation by SACs have been proposed: (1) The catalyzed persulfate self-decay pathway. Some studies have reported the formation of ^1^O_2_ through the slow self-dissociation of PMS (0.2 M^−1^s^−1^) (Equation (16)) [119]. (2) The metal-Nx structure-mediated pathway. The metal-Nx structure absorbs electrons from the surrounding C atoms, rendering the C atoms positively charged. This facilitates the adsorption of negatively charged PMS molecules on the positively charged C atoms, followed by generating ^1^O_2_, similar to PMS self-dissociation under alkaline conditions (Figure 4b) [64]. (3) The SO_5_^•−^ radical-involved oxidation pathway. Metal sites tend to adsorb the terminal O atom of sulfate, then releasing an electron from PMS to form SO_5_^•−^ (Equation (17)) [42,113,120]. Subsequently, the self-reaction of SO_5_^•−^ occurs quickly to generate ^1^O_2_, because of its high reaction rate (~2 × 10^8^ M^−1^s^−1^) and low activation energy (7.4 ± 2.4 kcal mol^−1^) (Equations (18) and (19)) [120]. Monoatomic Cu anchored on Ti_3_C_2_T_x_ MXene has been developed for efficient and selective ^1^O_2_ generation via PMS activation via the above-mentioned route (Figure 4c) [42]. Additionally, free SO_5_^•−^ can be captured by water to generate ^1^O_2_ (Equation (20)) [91].

HSO_5_^−^ + SO_5_^2−^ → SO_4_^2−^ + HSO_4_^−^ + ^1^O_2_ (16)

HSO_5_^−^ − e^−^ → SO_5_^•−^ + H^+^ (17)

SO_5_^•−^ + SO_5_^•−^ → S_2_O_8_^2−^ + ^1^O_2_ (18)

SO_5_^•−^ + SO_5_^•−^ → 2SO_4_^2−^ + ^1^O_2_ (19)

2SO_5_^•−^ + H_2_O → 2HSO_4_^−^ + 1.5 ^1^O_2_ (20)

(4) The continuous dissociation pathway. The PMS is initially adsorbed on the catalyst surface and then dissociated into surface-adsorbed SO_4_* and OH* moieties. The OH* is adsorbed onto metal-N-C sites. After the removal of H from OH* with thermodynamically forming O*, ^1^O_2_ is selectively generated (PMS→OH*→O*→^1^O_2_ (Figure 4d) [93,94,121]. (5) The O_2_^•−^ oxidation pathway. Oxygen molecular in water serves as an oxidant, donating electrons to catalysts and being reduced to O_2_^•−^ [121]. O_2_^•−^ is regarded as a precursor for ^1^O_2_ formation via the proton-promoted disproportionation shown in Equations (21)–(23) [95,96,122].

2O_2_^•−^ + 2H_2_O → ^1^O_2_ + H_2_O_2_ + 2OH^−^ (21)

O_2_^•−^ + H^+^ → HO_2_^•^ (22)

O_2_^•−^ + HO_2_^•^ → ^1^O_2_ + HO_2_^−^ (23)

In the case of PDS, despite its symmetrical charge distribution, electron transfer from PDS to the single atom upon adsorption results in its asymmetrical electron structure. The bond dissociation energy of S-O is lower than that of O-O bond, indicating the S-O bond is more susceptible to cleavage to form a PMS-like molecule that is further activated by SACs to produce ^1^O_2_ (Figure 4e) [97]. Additionally, the polar structures such as Fe-N trigger the polarization of the O-O bond in PDS, generating a surface intermediate that self-decomposes to produce ^1^O_2_ (Equations (24) and (25)) [71]. Furthermore, ^1^O_2_ can be also produced during the process of electron donation from PDS to electrophilic C=O groups of SA Co/CN catalyst [98].

N-Fe + SO_3_^−^-O-O-SO_3_^−^ → N-Fe≡SO_3_^−^-O(σ-)-O(σ+)-SO_3_^−^ (24)

2N-Fe≡SO_3_^−^-O(σ-)-O(σ+)-SO_3_^−^ + 2H_2_O → 2N-Fe + ^1^O_2_ + 4SO_4_^2−^ + 4H^+^ (25)

### 3.3. High-Valent Metal Species

High-valent metal species such as Fe(IV)=O [72,99], Mn(IV)=O [100], and Cu(III) [101] have been revealed as the dominant oxidative species in SAC-activated persulfate systems. To verify the role of high-valent species, DMPO was applied to trap them, yielding a signal of 5,5-dimethyl-2-pyrro-lidone-N-oxyl (DMPOX) for EPR analysis [72,90]. Furthermore, dimethyl sulfoxide (DMSO) is assumed to be oxidized to DMSO_2_ by high-valent species, and thus DMSO is always used as a scavenger for high-valent species [95]. Peng et al. analyzed DMSO and DMSO_2_ concentrations via gas chromatography-mass spectrometry (GC-MS) in the Fe-N-C/PMS system [84], observing a decrease in DMSO content, while an initial increase within 5 min and then a decrease is seen in DMSO_2_. This suggests an initial oxidation of DMSO to DMSO_2_ by Fe(IV)=O within the first 5 min, followed by the continuous decomposition of DMSO_2_. 

Similarly, methylphenyl sulfoxide (PMSO) is also explored as a quencher, undergoing oxidation by high-valent metals to methylphenyl sulfone (PMSO_2_) via an oxygen atom transfer process (Equation (26)). However, this detection method might be inapplicable at neutral pH due to the PMS-mediated oxidation of PMSO to PMSO_2_ [79,123]. Of note, the rapid reaction rates of PMSO with SO_4_^•−^ and ^•^OH without the generation of PMSO_2_ ($k^{•}$_OH/PMSO_ = 3.61 × 10^9^ M^−1^s^−1^ and $k_{{SO}_{4}^{•-}}$_/PMSO_ = 3.17 × 10^8^ M^−1^s^−1^) (Equations (27) and (28)) may interfere with the detection of high-valent metals [124].

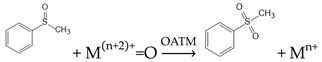
(26)

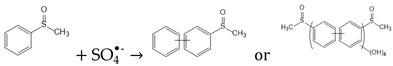
(27)

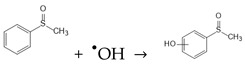
(28)

**Figure 5:**
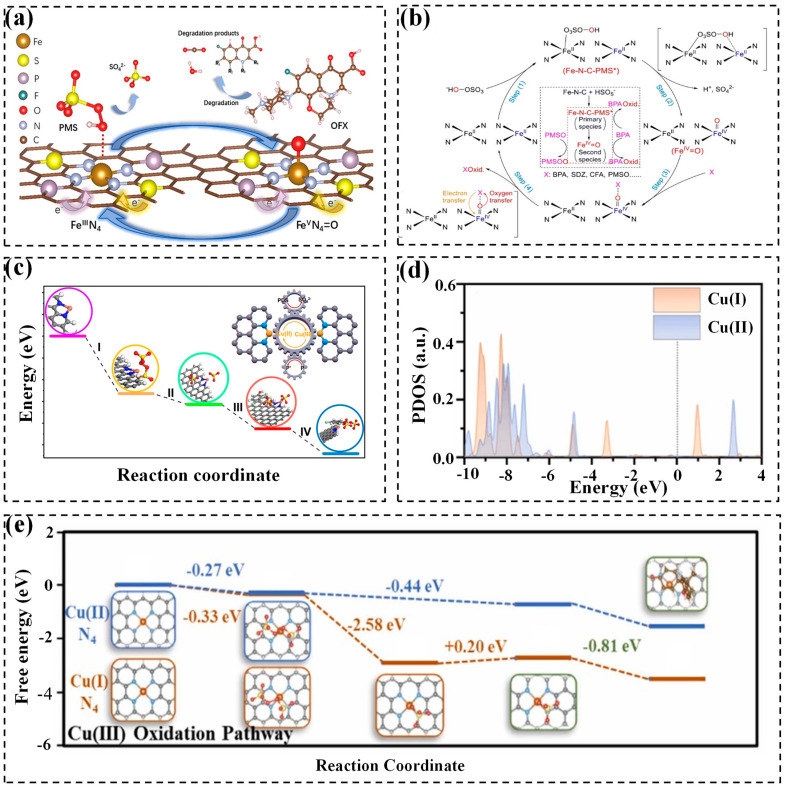
(**a**) The mechanism of OFX removal in the Fe_SA_-NPS@C/PMS system [102]. Copyright 2023 Elsevier. (**b**) The Fe^IV^=O generation process in the Fe-N-C/PMS system [125]. Copyright 2023 Wiley-VCH. (**c**) Activation pathway of PDS on Cu_SA_-NC catalyst [101]. Copyright 2022 American Chemical Society. (**d**) Partial density of states (PDOS) of Cu atoms in Cu(I)N_4_ and Cu(II)N_4_ sites [126]. Copyright 2024 Elsevier. (**e**) Activation pathways of PDS at Cu(I)N_4_ and Cu(II)N_4_ sites [126]. Copyright 2024 Elsevier.

The formation pathways of high-valent metals are summarized as follows. In PMS-based systems, metal sites like Fe(III) can coordinate with the O atom of PMS to form surface complexes ([Fe(III)OOSO_3_]^+^), which subsequently undergo heterolysis of the O-O bond, producing high-valent metal-oxo species like Fe(IV)=O and Fe(V)=O (Equations (29)–(31)) [72,95]. Alternatively, PMS can directly capture electrons from active sites (i.e., Fe-N_x_) to generate high-valent metals (Equation (32)), which subsequently undergo reduction by organic pollutants (Figure 5a) [102,103]. 

The detailed formation of Fe(IV)=O has been further explored through DFT and activation energy (ΔG) calculations. Cheng et al. demonstrated that the Fe(IV)=O formation requires the accumulation of Fe-N-C-PMS*, facilitated by dual-site adsorption and thermodynamically favorable energy profiles [125]. The length of the adsorbed PMS molecule (4.2 Å) closely matches with the distance between monoatomic Fe (4~5 Å), facilitating the formation of a more stable dual-site adsorption configuration. Moreover, the lower ΔG associated with the 2Fe-N_4_ site is in favor of a thermodynamically stable formation of Fe(IV)=O on this site. Thus, Cheng et al. posited a mechanistic pathway where PMS initially adsorbs onto Fe-Fe sites with a certain distance of 4~5 Å (step 1). Afterwards, the hydroxy O in Fe(III)OOSO_3_ engages in coordination with an adjacent Fe(II)-N_4_ to form an inter-complex structure. This transformation is accompanied by the liberation of 2.27 eV of energy, which is harnessed to cleave the O-O and O-H bonds, ultimately resulting in the generation of Fe(IV)=O (step 2). Finally, the pollutant molecule is sequestered onto the O atom and undergoes electron loss to be degraded (steps 3 and 4) (Figure 5b). This groundbreaking study offers a new perspective on the mechanism underlying the generation of Fe(IV)=O in SAC-mediated pollutant degradation.

≡Fe(III) + HSO_5_^−^ → [Fe(III) OOSO_3_]^+^ + H^+^ (29)

[Fe(III) OOSO_3_]^+^ → ≡Fe(IV) = O + SO_4_^•−^ (30)

≡Fe(IV) = O + TC → products + ≡Fe(III)(31)

Fe(III)N_4_ + PMS → Fe(V)N_4_ = O + SO_4_^2−^ + H^+^ (32)

For PDS-based systems, transition metals are assumed to receive two electrons from PDS for generating high-valent metal [99]. In the Cu_SA_-NC/PDS system, two Cu(II)-N_2_ can donate 2e^−^ to PDS and be oxidized to 2Cu(III)-N_2_, as outlined in Equation (33). In step I, the PDS adsorbs on the Cu atom of Cu_SA_-NC and then the PDS molecule undergoes activation and subsequent dissociation to produce SO_4_^2−^ ions (step II). Subsequently, the contaminant adsorbs on Cu_SA_-NC (step III) and is oxidized by Cu(III)-N_2_ species to form the intermediate products (step IV) (Figure 5c) [101]. Pan et al. further distinguished the roles of active sites Cu(I)N_4_ and Cu(II)N_4_ in forming high-valent Cu(III)N_4_ in the SACu_30_@NC/PDS system. The results revealed that Cu(I)N_4_, due to its proximity to the Fermi energy level, exhibits higher reactivity with PDS (Figure 5d), facilitating the generation of Cu(III)N_4_. The free energy calculations support this finding. As depicted in Figure 5e, the total released energy of the five generation steps of Cu(III)N_4_ are counted as −3.52 eV and −1.52 eV for Cu(I)N_4_ and Cu(II)N_4_, respectively, indicating that the Cu(I)N_4_ more likely acts as the active site to initiate the formation of Cu(III)N_4_ [126].

2 Cu(II)-N_2_ + S_2_O_8_^2−^ → 2Cu(III)-N_2_ + 2SO_4_^2−^ (33)

### 3.4. Direct Electron Transfer

In contrast to the generation pathways of radicals, ^1^O_2_, and high-valent metals, in which persulfate abstracts electrons from metal sites to produce reactive species that further attack pollutants, persulfate can also abstract two electrons from pollutants in generating SO_4_^2−^ by utilizing surface metal sites as an electron transfer conduit, which is known as a direct electron transfer oxidation system [30]. 

**Figure 6:**
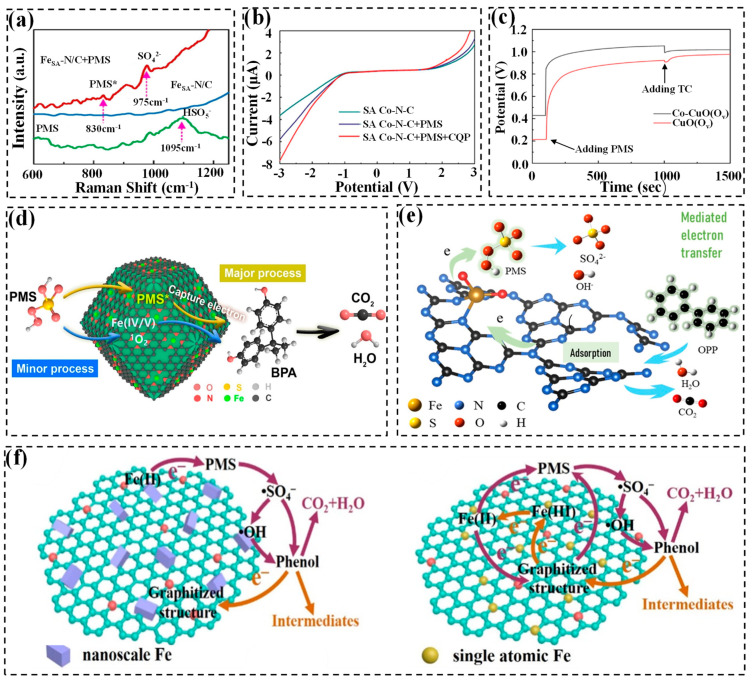
(**a**) In situ Raman spectra partial in different systems [44]. Copyright 2021 Elsevier. (**b**) LSV tests [82]. Copyright 2022 Elsevier. (**c**) The OCP measurements of a SAC with single Co atom dispersed on CuO [82]. Copyright 2023 Elsevier. (**d**) The mechanism of BPA removal in the Fe_SA_-N/C/PMS system [44]. Copyright 2021 Elsevier. (**e**) Electron transfer mechanism for OPP oxidation in the Fe/g-C_3_N_4_-activated PMS system [90]. Copyright 2021 Elsevier. (**f**) Activation mechanism of PMS by nano-Fe/MC and ISA-Fe/MC catalysts respectively [104]. Copyright 2021 Elsevier.

Multiple experiments can corroborate this mechanism: (1) the quenching agent experiments and EPR techniques rule out the involvement of radicals, ^1^O_2_, and high-valent metals [82,104]. (2) The Raman spectra show a peak of adsorbed peroxo species (i.e., PMS* at approximately 830 cm^−1^), demonstrating the attachment of PMS on the SACs’ surface (Figure 6a) [44]. (3) The linear sweep voltammetry (LSV) curves of the SAC/PMS and SAC/PMS/pollutant systems exhibits significant alteration compared to the bare SAC system. This can be attributed to the formation of a surface reactive complex over the catalyst, as well as the oxidation of contaminants via electron transfer (Figure 6b) [82]. (4) The current i-t curve presents a significant increase in current upon the injection of pollutants, indicating the generation of current flow from the pollutant or PMS to the surface of the SAC-loaded electrode [105]. (5) In open-circuit potential (OCP) measurements, the potential of the catalyst rose rapidly after adding PMS, suggesting the generation of a surface PMS complex with a high redox potential. The potential decrease in the presence of contaminants is due to the surface PMS complex’s decomposition after accepting electrons from contaminants (Figure 6c) [127]. 

The electron transfer process for pollutant degradation can be summarized as follows: Firstly, PS is absorbed to the surfaces of SAC-based catalysts and metastable complexes (M-PS*) can be formed. Subsequently, the oxidizability of the PS increases due to the partially altered electron distribution, which helps to obtain electrons from pollutants and degrade the pollutants, along with the persulfate decomposition [128]. Taking Fe-based SACs as an example, Yang et al. have developed Fe-doped FePc@ZIF-8 (Fe_SA_-N/C) to activate PMS for BPA degradation via an electron-transfer regime [44]. BPA is adsorbed onto the Fe-Nx site by a “donor-acceptor complex” mechanism to form a nonradical PMS* intermediate during the reaction, which facilitates the electron transfer between PMS and Fe_SA_-N/C (Figure 6d). Furthermore, Duan et al. proposed a novel understanding regarding the mechanism of mediated electron transfer. Specifically, the atomically dispersed Fe on g-C_3_N_4_ (SA Fe-CN) has been employed in activating PMS to remove o-phenylphenol (Figure 6e) [90]. The divergent electron density of the triazine ring transfers to Fe-O_2_ bond, which promotes the triazine ring to accept electrons from organic pollutants. Meanwhile, Fe-O_2_ donates an electron to PMS after bonding with a PMS molecule. Of note, the O-O bond of PMS with electron depletion does not instantly break and facilitates the electron transfer from Fe-O_2_ to PMS after acquiring electrons from contaminants, enabling the continuous decomposition of pollutants.

Li et al. provided a detailed explanation of the mechanism differences between the SA-Fe/MC/PMS system dominated by direct electron transfer and the nano-Fe/MC/PMS system dominated by free radicals (Figure 6f) [104]. In particular, in the nano-Fe activation system, the Fe(II) of nanoscale iron particles can donate one electron to PMS, generating reactive radicals. In contrast, in the SA-Fe activation system, electrons from organics transfer to sp^2^-hybridized C in graphitized structures, with phenol being oxidized to intermediates. In this process, single-metal Fe serves as an electron transfer conduit between PMS and organic pollutants, suggesting a mechanism of direct electron transfer oxidation in the SA-Fe/MC/PMS system. It should be noted that the inherent delocalized p electrons of sp^2^-hybridized C can also donate electrons to the Fe(III) over the biochar sheet surface, thereby forming Fe(II) for the subsequent cycle of activation. 

## 4. Influencing Factors

### 4.1. Oxidant Concentration

The PS concentration significantly impacts the removal efficiency of contaminants. In general, an increase in the concentration of peroxides leads to an enhancement in contaminant removal [84]. Li et al. reported that the first-order kinetic constant (*k*) of phenol elimination elevated from 0.329 min^−1^ to 2.788 min^−1^ as the PMS concentration increased from 0.25 g/L to 1.0 g/L [104]. Peng et al. further found a linear correlation of *k* with initial PS concentration [82], which was attributed to the fact that increasing PS generated more reactive species to degrade pollutants. However, there exists an optimal PS concentration beyond which the degradation rate plateaus or even declines. This is because excessive oxidant content probably inhibits the catalytic performance due to the self-quenching reaction among SO_4_^•−^ (Equation (34)) and the reaction with excess persulfate (Equations (35) and (36)) in the radical-based oxidation pathways [40]. In addition, in nonradical oxidation processes, high oxidant concentrations may occupy the surface active sites of the catalyst, thus hindering the efficient degradation of pollutants [71].

SO_4_^•−^ + SO_4_^•−^ → S_2_O_8_^2−^ (34)

SO_4_^•−^ + S_2_O_8_^2−^ → SO_4_^2−^ + S_2_O_8_^•−^ (35)

SO_4_^•−^ + HSO_5_^−^ → SO_5_^•−^ + SO_4_^2−^ + H^+^ (36)

### 4.2. Catalyst Dosage

Catalyst dosage is a pivotal operating parameter that dictates the rate of pollutant degradation. Generally, the catalytic performance will be enhanced as the catalyst dose increases [40], since more active sites can be available for PS activation, enhancing the formation of reactive species for degrading contaminants. Li et al. demonstrated that by elevating the catalyst dosage from 0.025 g/L to 0.05 g/L in the ISA-Fe/MC/PMS system, the *k* value for contaminant degradation soared from 0.132 min^−1^ to 1.095 min^−1^ [104]. Additionally, a linear correlation between *k* and the initial catalyst dosage was also observed in the nonradical pathways [84]. However, once sufficient active sites of the catalyst for PS activation are present, further increasing the catalyst dosage may not lead to substantial enhancements in the degradation efficiency of pollutants [82]. 

### 4.3. Solution pH

The solution pH always exerts a profound effect on the degradation rate of contaminants. Generally, the electrostatic interaction among the pollutant, catalyst, and PS would be affected by the pH value, as persulfate is negatively charged during reactions, while the surface charge of catalysts varies with pH [40,44,71,84]. For instance, the zero charge point of Fe-based SACs is approximately 5.0, implying a positively charged surface of the catalyst under a pH below 5.0 and a negatively charged surface under a pH above 5.0. Consequently, at higher pH values, electrostatic repulsions may arise between PMS and the catalyst surface, impeding the electron transfer and decreasing the reaction rate [77]. 

**Figure 7:**
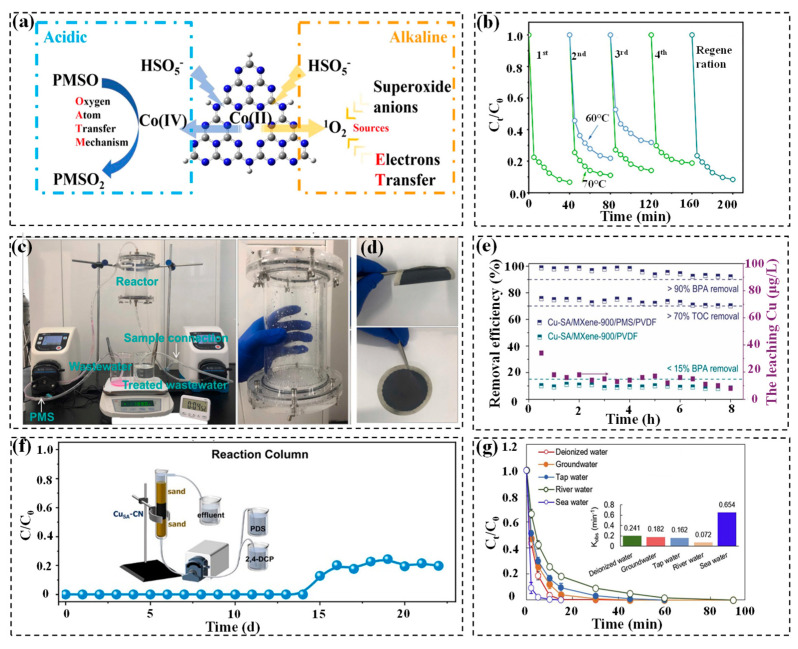
(**a**) The mechanism of BPA removal in acidic and alkaline environments [129]. Copyright 2022 Elsevier. (**b**) Cycling test of SA Fe-g-C_3_N_4_ catalyst [95]. Copyright 2022 Elsevier. (**c**) The diagram of experimental setup [42]. (**d**) The photo of membrane device [42]. (**e**) The removal of BPA and the leaching Cu ions in a continuous reactor [42]. Copyright 2023 Elsevier. (**f**) 2,4-DCP concentration in CuSA-NC-filled column [101]. Copyright 2022 American Chemical Society. (**g**) Removal of NPX by the SA Co-N/C-activated PMS system in various water matrices [81]. Copyright 2021 Elsevier.

Side reactions also affect the deterioration rates with pH variations. Under an acidic condition, H^+^ ions compete with the catalyst for bonding to negatively charged oxidants and active sites [71,106]. At pH 10, OH^−^ ions can react with the active species SO_4_^•−^ [91]. In alkaline environments, PMS may interact with high-valent Fe substances to hinder the degradation of pollutants [95]. Besides, the solution pH could influence the generation of reactive species. Wang et al. developed a cobalt-based SAC supported on g-C_3_N_4_ for efficient BPA removal in a wide pH range. Their findings revealed that Co(IV)=O is the dominant reactive species in acidic conditions, whereas ^1^O_2_ dominates the oxidation of pollutants at an alkaline pH (Figure 7a) [129]. Nevertheless, the specific mechanisms underlying the effect of pH on reaction pathways require further investigation. 

Interestingly, pH exhibits negligible effects on pollutant degradation in certain systems [64,92,120]. Duan et al. demonstrated that the SAFe-CN-activated PMS system possessed a good adsorption and catalytic oxidation performance across a broad pH range of 3–11 [90]. Similarly, Zuo et al. developed a sandwich structural catalyst with atomic Fe metal (C_3_N_4_-Fe-rGO) that exhibited excellent catalytic performance at all pH values (0–14) [110]. This can be attributed to the double-layer confined structure, which effectively prevents Fe atom agglomeration and acid leaching even under extreme pH conditions.

### 4.4. Inorganic Anions and Organic Matters 

Real water is a complex system that typically contains diverse inorganic substances (i.e., HCO_3_^−^, Cl^−^, and HPO_4_^2−^) and organic species (i.e., humic acids (HA)). These compounds engage in multifaceted interactions, either competing with persulfate for active site occupation or directly reacting with them. As a result, they have either a promotion or inhibitory influence on the degradation of organic contaminants. 

HCO_3_^−^, a ubiquitous inorganic ion in aqueous environments, exerts a profound effect on contaminant removal. It is generally considered that HCO_3_^−^ can consume SO_4_^•−^ and ^•^OH, thereby inhibiting the degradation process [71]. However, in a few cases, the resulting CO_3_^•−^ radicals (Equations (37) and (38)) might in turn facilitate the degradation of N/O-functionalized contaminants like oxytetracycline [107]. Furthermore, the presence of HCO_3_^−^ shifts the pH of reactive systems towards neutrality or alkalinity, further affecting the degradation efficiency in response to solution pH variations [82,130].

HCO_3_^−^ +^•^OH→ CO_3_^•−^ + H_2_O (37)

HCO_3_^−^ + SO_4_^•−^ → H^+^ + CO_3_^•−^ + SO_4_^2−^ (38)

Cl^−^, one of the most prevalent anions in water matrices (i.e., present at approximately 1.0 mM in freshwater and 100 mM in industrial effluent), exerts a dual impact on degradation efficacy, depending on its concentration. A low concentration of Cl^−^ can inhibit the pollutant removal by forming Cl^•^ with low redox potential through reactions with ^•^OH and SO_4_^•−^ (Equation (39)) [104]. However, at elevated concentrations, it promotes the degradation of pollutants via the generation of long-lived HOCl species [84]. Notably, some studies have reported opposing effects, with observing an enhancement in aniline degradation due to HOCl_2_^•−^ generation at Cl^−^ concentrations of 0~10 mM (Equations (40) and (41)) [40], whereas Peng et al. found a minimal impact on chloroquine phosphate degradation in the SAC-Co/PMS system over the same concentration range, which might be attributed to nonradical-dominated oxidation pathways [82].

Cl^−^ + ^•^OH/SO_4_^•−^→ OH^−^/SO_4_^2−^ + Cl^•^ (39)

Cl^•^ + Cl^−^ → Cl_2_^•−^ (40)

Cl_2_^•−^ + H_2_O → HOCl_2_^•−^ + H^+^ + SO_4_^•−^ (41)

With regards to HPO_4_^2−^, its dual role in the oxidation process has also been reported. HPO_4_^2−^ not only reacts with ^•^OH and SO_4_^•−^ to generate H_2_PO_4_^•^ with a low oxidation potential [104], but also interferes with the absorption between PS and the catalyst [52]. Peng et al. reported that low concentrations of HPO_4_^2−^ (0~5 mM) can promote PMS decomposition in the carbon-based SAC/PMS system for enhancing chloroquine phosphate degradation, while a high concentration (10 mM) leads to a slight inhibition of pollutant removal, likely due to catalyst surface deactivation through the adsorption of HPO_4_^2−^ [82]. Natural organic matter (NOM) is ubiquitous in various water matrices and its influence on the removal of contaminants should not be underestimated. For instance, humic acid (HA), a typical NOM, possesses many hydroxyl and carboxyl groups which tend to combine with surface active sites of catalysts, thus hindering the absorption and activation of persulfate [44,71,72]. Additionally, HA can react with ROS such as ^1^O_2_ and high-valent metals, interfering with the elimination of contaminants in water [92,123]. 

### 4.5. Operando Stability

Despite SACs demonstrating remarkable performance in contaminant degradation, their stability during reactions often remains overlooked. SACs may suffer active site loss due to the damage of local coordination and support, resulting in compromised performance. Hence, ensuring SAC stability in practical reaction conditions is paramount. 

Recycling tests are conducted to assess the stability and reusability of SACs. Peng et al. examined the reusability of SAC Fe-g-C_3_N_4_ for tetracycline (TC) degradation. TC was removed by >90% in the first and second cycles, with a ~12% decline after four cycles. The catalysts that were subjected to a drying process at a temperature of 70 °C outperformed those at 60 °C, probably owing to the better removal of surface coverings at higher temperatures (Figure 7b). Of note, the catalytic performance of Fe-g-C_3_N_4_ was restored through thermal regeneration at 350 °C for 3 h under a N_2_ atmosphere. This thermal treatment is considered to remove intermediate products adsorbed on the active center and the edge of the catalyst [95]. 

Long-term experiments using continuous-flow devices (Figure 7c,d) have always been applied to further validate SAC durability, maintaining >90% removal and >70% mineralization of BPA over 8 h in the Cu-SA/MXene/PVDF/PMS system (Figure 7e) [42]. In this simple continuous-flow reactor, Cu-SA/MXene was thoroughly distributed and subsequently filtered onto a polyvinylidene difluoride (PVDF) membrane. Similarly, Gao et al. set up a flow reactor with Co SAC distributed on PTFE membrane. The result indicated that the nitenpyram degradation maintained 100% over 10 h [131]. However, the cost of membrane materials is high and they are prone to being clogged or deactivated by impurities and degradation intermediates, which is detrimental to long-term catalytic degradation [132]. Another continuous-flow device is the catalyst-filled column, which can significantly improve the contact of reactants [132]. A catalyst-filled column was sequentially filled with quartz sand, catalyst, and quartz sand. A mixture of pollutant and PS flowed into the bottom of the column with the quartz sand layer and then fully reacted with the catalyst, removing pollutants in the catalyst layer, and finally the treated solution flowed out from the top of the column. Li et al. demonstrated an excellent stability of CuSA NC for 2,4-DCP degradation over 14 days by a continuous flow reactor comprising a catalyst-filled column (Figure 7f), emphasizing its primary oxidation decomposition mechanism over adsorption [101]. The monitoring of metal ions’ dissolution is another way to assess the stability of a catalyst. Liao et al. developed individual Co anchored on a 2D carbon nanoplate catalyst to activate PMS for degrading acetaminophen. All concentrations of leaching Co^2+^ at pH 3~9 were significantly lower than the emission standard (1 mg/L) of China, indicating the stability of SA-Co CNPs in a wide range [92]. 

### 4.6. Real Water

Recent studies have delved into the viability of utilizing SACs for the elimination of pollutants in natural water sources, wastewater effluents of water reclamation facilities, and leachate from landfills, respectively. Qi et al. developed a SA Co-N/C catalyst to degrade naproxen (NPX) via PMS activation in distinct water matrices (Figure 7g). The removal of NPX in tap water slightly diminishes owing to the Cl^−^ ions’ quenching effect on the free radicals. As for river water, the complex background constituents (i.e., organic matter and anions) impede the oxidation rate of NPX, whereas abundant the Cl^−^ ions in seawater enhance NPX degradation owing to the generation of HOCl. Despite interference from real water constituents, NPX is ultimately fully degraded across multiple aquatic environments [81]. In addition to surface water, the SA Fe-OCN-activated PMS system was also employed in wastewater effluents of a water reclamation facility, aiming at extending its practical application. The system exhibited excellent catalytic performance for phenol degradation in the above complex water [123]. Furthermore, the leachate with a COD of 71.3 mg/L was used as a target body in the Fe-N_3_/C/PMS system. Results indicated that 87% of COD could be removed during 40 min of reaction, demonstrating the great application potential of the Fe-N_3_/C/PMS system [133]. Briefly, in actual water treatment process, the SACs based PS-AOP technology can be flexibly integrated with existing wastewater treatment processes to form an efficient system [134]. Importantly, conventional treatments only change the phase of these organic contaminants and do not transform them into benign compounds, while this technology has demonstrated the potential for the rapid degradation of toxic contaminants in wastewater [8]. Furthermore, it can be used as a post-treatment to biological treatments due to the capacity of AOPs to convert non-degradable compounds into more biodegradable products which benefit further biological treatment [135].

## 5. Pollutants Removal 

The elimination of a broad spectrum of pollutants by SAC-activated persulfate has garnered considerable attention in recent years, including azo dyes (i.e., acid orange 7 (AO7), rhodamine B (RhB), methyl orange (MO) et al.) [106,136], phenolic compounds (i.e., 4-chlorophenol (4-CP), bisphenol (BPA), diclofenac (DCF), chlorophenol (CP), 4-methoxy-phenol (MOP) et al.) [89,120], pharmaceuticals (i.e., sulfamethoxazole (SMX), sulfadiazine (SDZ), aspirin (ASA), naproxen (NPX), paracetamol (PCM), ciprofloxacin (CIP), metronidazole (MNZ), anybenzoic acid (HBAc) et al.) [81,94,97,108,123], and even bacteria such as *E. coli* [137,138]. 

**Figure 8:**
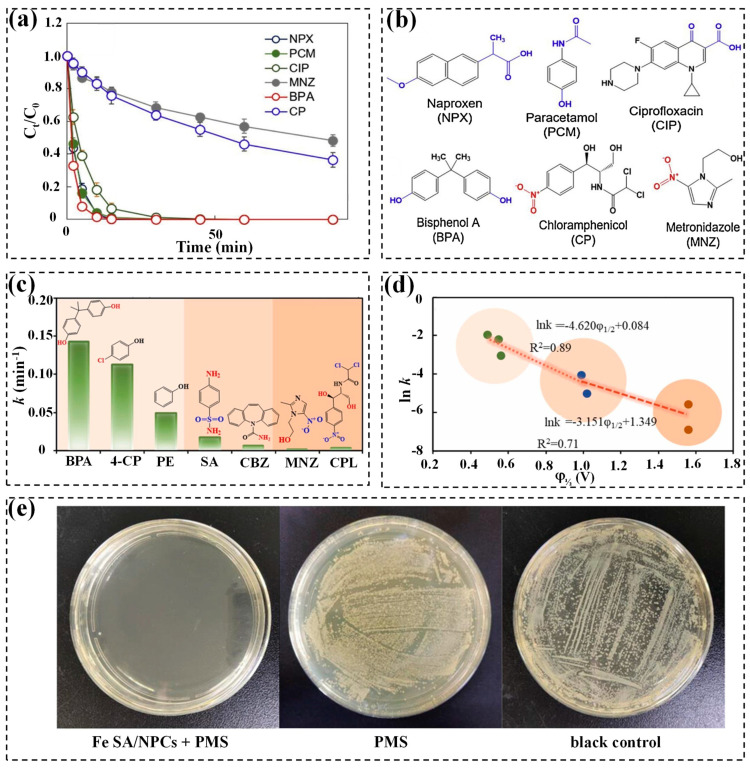
(**a**) Removal of various pollutants in the SA Co-N/C/PMS system [81]. (**b**) Structures of a variety of pollutants [81]. Copyright 2021 Elsevier. (**c**) *k* values of contaminant removal in the SACu@NC/PDS system [126]. Copyright 2024 Elsevier. (**d**) Relationship between φ_1/2_ values of pollutants and *k* in the SACu@NC/PDS system [126]. Copyright 2024 Elsevier. (**e**) Colony formation assays for *E. coli* cultures treated with different catalytic systems [137]. Copyright 2022 Wiley-VCH.

The removal performance of organic contaminants tends to correlate with the electron-donating or -withdrawing nature of their substituents, as well as their ionization potential. Electron-donating groups like hydroxyl (-OH) and acylamde (-CO-NH_2_) are easily oxidized due to their low ionization potential. On the contrary, electron-absorbing groups, including carboxyl (-COOH) and nitro groups (-NO_2_), are more difficult to oxidize [108]. Figure 8a illustrates the removal of various pollutants in the SA Co-N/C/PMS system, where MNZ and CP, containing electron-withdrawing -NO_2_ groups, exhibit a slower degradation efficiency (40~60% within 90 min), whereas -OH-bearing contaminants like PCM and BPA are more susceptible to being oxidized, with 100% removal within 90 min [81]. The chemical structures of these pollutants are listed in Figure 8b. Of note, both CIP and NPX still undergo rapid degradation, despite containing electron-withdrawing -COOH groups. Pan et al. proposed that the degradation of pollutants in the SA Cu30@NC/PDS system correlated with their redox potentials, as evidenced by the linear correlation between the removal rate of various pollutants and their half-wave potentials (φ_1/2_) (Figure 8c,d). This result highlights the importance of the redox capacity of pollutants in degradation kinetics [126]. Additionally, the concentration of organic contaminants may also affect the degradation performance. As shown in Table 3, a wide range of pollutant content (0.93~50 mg/L) could be eliminated by a SAC-activated PS system. However, the pollutants’ removal depends on the dosages of PS and catalyst in various systems. Zhang et al. demonstrated a negative correlation between the removal rate of carbamazepine (CBZ) and dosage of CBZ. Specifically, an increase in CBZ dosage (from 2.5 mg/L to 10 mg/L) negligibly impacted the degradation efficiency, whereas the removal of CBZ dramatically dropped as the CBZ concentration was over 20 mg/L, which might be ascribed to the occupation of active sites by excessive CBZ molecules [139]. Moreover, SAC-activated persulfate systems have demonstrated their vast potential in bacterial inactivation. Yang et al. reported the complete eradication of *Escherichia coli* (*E. coli*) within 5 min using the Fe SA/NPCs/PMS system (Figure 8e), whereas PMS alone showed a minimal bactericidal effect [137]. Similarly, Zhou et al. demonstrated efficient *E. coli* inactivation in the Ru/NiFe-LDH/PMS system [138], underscoring the need for further exploration to extend this technology to other bacterial species.

## 6. Conclusions and Prospect

Over the past 10 years, the application of SACs has garnered significant attention for pollutant removal in aqueous solutions. This review delves into the supports and synthesis methods of SACs. The primary reactive species generated by SAC-activated persulfate systems and their formation mechanisms are elaborated in detail. The influencing variables of the operating conditions and the impact of actual water quality are further examined. The removal of diverse pollutants, including dyes, phenolic compounds, pharmaceuticals, and bacteria is also highlighted. Although SAC-activated persulfate systems have great application prospects in wastewater treatment, there are some challenges in their practical applications.

(1) The high surface free energy of SACs probably causes atom agglomeration, resulting in the generation of metal nanoparticles and nanoclusters that hinder catalytic activity and waste precious metal resources. To avoid single-atom aggregation, many researchers focus on the preparation of low metal-loaded SACs; however, the low active sites highly restrict the catalytic oxidation performance in practical applications. Thus, it is crucial to design SACs with a suitable atomic load that balance their economic benefits and pollutant degradation efficiency.
(2) Recycling SACs from aqueous suspensions is still a difficult task due to their small particle sizes. To improve the capacity of recovery and reusability of SACs, SACs have been coated into porous carbon material surfaces, such as carbon felt, carbon cloth, and functional cotton fiber, which can serve as membrane filters in water treatment. However, the impact of the specific surface area and stability of different loaded carbon materials on catalytic activity is rarely reported, deserving further exploration.
(3) Real aquatic media typically contain diverse background components which can deplete reactive oxygen species or lead to catalyst surface contamination, hence impacting the overall effectiveness of the oxidation process. Thus, developing SACs with exceptional stability, reactivity, and selectivity is vital for their optimal performance in complex environments.
(4) Scaling up laboratory models to large-scale pilot experiments remains a bottleneck for SAC-based technology. Given the diversity of pollution scenarios, it is necessary to design distinct reactors tailored for utilizing SACs in pollutant degradation. It is recommended to conduct more field or on-site experiments in future research.
(5) The design and synthesis of SACs demands a considerable investment of time, expertise in chemistry, dedicated effort, and a process of repeated testing. To streamline this process, quantitative structure-activity relationship (QASR) data analysis and machine learning (ML) could be employed in the future to establish correlations between catalytic performance and various factors such as catalyst properties and operational parameters. This approach could significantly expedite the process of catalyst screening and discovery.

## Figures and Tables

**Table 1:** Summary of the recently published reviews on the AOP systems catalyzed by SACs.

Title	Review Scopes	Knowledge Gaps	Ref.
Outlook on single atom catalysts for persulfate-based advanced oxidation	(1) Overview of fundamental research with theoretical simulation and in situ characterization techniques (2) Overview on various oxidation mechanism	(1) The effects of process parameters are not included (2) Various pollutants removal is not highlighted	[30]
Nitrogen-doped carbon-based single-atom Fe catalysts: Synthesis, properties, and applications in advanced oxidation processes	(1) Overview of carbon-based Fe-SACs for the organic pollutants removal in water (2) Summary of the synthesis strategies of SACs (3) Investigation on the reaction mechanisms of Fe-SAC-based AOPs	(1) Lack of other metal based-SACs’ descriptions (2) The effect of structural characteristics of pollutants on removal performances has not been analyzed	[31]
Single-atom catalysis in advanced oxidation processes for environmental remediation	(1) The synthetic strategies, characterization, and DFT calculation of carbon-based SACs were investigated (2) The mechanisms of pollutant removal over carbon-based SACs were discussed	(1) There is no investigation about metal supported-SACs (2) The mechanism of active species generation is not described in detail	[32]
Recent progress on single-atom catalysts in advanced oxidation processes for water treatment	(1) The classification of SAC-mediated AOPs for the degradation of pollutants, including photocatalysis, electrocatalysis, Fenton-like reactions, persulfate oxidation and multi-technology coupling reactions (2) The formation mechanism of ROS was discussed	(1) PS-based AOP technology was not described in detail (2) The impact of key factors is not included	[33]
Single atom catalysts for degradation of antibiotics from aqueous environments by advanced oxidation processes: A review	(1) The coordination environment of SACs was discussed (2) An overview of diverse SAC-mediated AOPs systems for antibiotics degradation, encompassing photocatalysis, electrocatalysis, H_2_O_2_/persulfate based AOPs (3) Impact of key factors was discussed	(1) The formation mechanism of reactive oxygen species is insufficient (2) The degradation of various pollutants is not included	[34]
Single atom is not alone: Metal-support interactions in single-atom catalysis	(1) Interactions between the metal active sites and the support matrix were investigated (2) Discussion of current challenges and future opportunities for SACs	(1) Lack of comprehensive overview on the generation mechanism of active species (2) The effects of process parameters are not considered	[35]
Microenvironment engineering of single-atom catalysts for persulfate-based advanced oxidation processes	(1) Summary of microenvironment engineering of SACs for persulfate activation (2) Strategies for regulating and characterizing the microenvironment are illustrated	(1) The formation mechanism of reactive oxygen species is insufficient (2) The impact of key factors is not included (3) The effect of structural characteristics of pollutants on their removal performances is not discussed	[36]

**Table 2:** Summary of different support materials for single-atom hosts.

Supports	Structure	Surface Area	Characteristics	Ref.
Metal hydroxide (NiFe-LDH)	Composed of positively charged metal hydroxide layers and exchangeable anions filling between the layers	62.8 m^2^/g	(1) The interlayer anions in LDHs can be regulated in terms of both species and quantity (2) Positively charged and hydroxyl functional groups on surface of LDHs	[46]
metal sulfide (MoS_2_)	Composed of two layers of sulfur atoms sandwiching a middle layer of molybdenum atoms	5~20 m^2^/g	(1) Highly visible light absorption (2) Effective large surface area charge activated surface (3) Low toxicity	[47]
Mxene (Ti_3_AlC_2_)	A layered composite material, composed of alternating arrangements of one layer of titanium carbide (TiC) and two layers of aluminum (Al)	5~22 m^2^/g	(1) High electrical conductivity (2) Superior corrosion resistance	[48]
g-C_3_N_4_	A novel type of nitrogen-doped carbon materials with a two- or three-dimensional structure, composed of lamellar hexagonal building blocks of triazine units, forming a lamellar π-conjugated structure consisting of sp^2^C and sp^2^N	12~113 m^2^/g	(1) Excellent photoelectric performance (2) Strong thermal stability	[49]
MOF (ZIF-8)	A nested three-dimensional porous network structure, where zinc ions and 2-methylimidazole alternate in arrangement to form a zeolite-like framework	1340 m^2^/g	(1) Good chemical and thermal stability (2) Metal nodes and organic ligands serving as catalytic sites (3) High selectivity and sensitivity	[50]
COF (TaPa)	A two-dimensional layered structure where organic monomers are connected through covalent bonds	984 m^2^/g	(1) Good chemical and thermal stability (2) Porosity (3) Selective catalysis (4) Optical properties	[51]
Biomass-based materials (lignin)	The three phenylpropanoid units, namely guaiacyl propane, syringyl propane, and p-hydroxyphenyl propane, are interconnected through ether bonds and carbon-carbon bonds to form a biological polymer with a three-dimensional network structure	1400~1600 m^2^/g	(1) Easily biodegradable (2) Environmentally friendly	[52]

**Table 3:** The summary of the reactive species in the SAC-activated persulfate systems.

Catalyst	Oxidant	Pollutant	Other Condition	Reactive Species	Detection Method	Degradation	Ref.
Fe-MoS_2_ (0.2 g/L)	PMS (0.3 g/L)	Rhodamine b (20 mg/L)	pH (6.0), T (25 °C)	SO_4_^•−^, ^•^OH, O_2_^•−^	EPR, radical scavenger experiments	100% (3 min)	[39]
Fe_x_Mo_1–x_S_2_ (0.1 g/L)	PDS (1 mM)	Aniline (10 μM)	pH (4.0), -	SO_4_^•−^, ^•^OH	EPR, radical scavenger experiments	100% (20 min)	[40]
Cu-SA/MXene (0.5 g/L)	PMS (2.0 mM)	Bisphenol A (10 mg/L)	pH_0_ (7), T (25 °C)	^1^O_2_	EPR, radical scavenger experiments	100% (10 min)	[42]
FeSA-N/C-20 (0.15 g/L)	PMS (0.4 g/L)	Bisphenol A (20 mg/L)	pH (6.5), -	Direct electron transfer, ^1^O_2_	chemical quenching experiments, EPR, in situ Raman spectra, electrochemical analysis	100% (20 min)	[44]
FeCNx (0.05 g/L)	PMS (0.15 mM)	Bisphenol A (0.088 mM)	pH_0_ (6.5), T (25 °C)	catalyst-PMS *, ^1^O_2_, SO_4_^•−^	EPR, electrochemical analysis	94% (5 min)	[64]
FeSA-N-C (0.15 g/L)	PMS (0.4 g/L)	Bisphenol A (20 mg/L)	pH (6.5), -	Fe(IV)=O, ^1^O_2_, SO_4_^•−^, ^•^OH	EPR, scavenger experiments	99% (15 min)	[72]
Fe@COF (0.1 g/L)	PMS (0.65 mM)	Orange II (20 mg/L)	-, T (25 °C)	^1^O_2_, O_2_^•−^	EPR, scavenger experiments	100% (45 min)	[77]
SA-Mn-NSC (0.2 g/L)	PMS (1 mM)	Enrofloxacin (10 mg/L)	pH (6.8), T (25 °C)	Mn(V), ^1^O_2_, SO_4_^•−^, ^•^OH, O_2_^•−^	EPR, scavenger experiments	100% (10 min)	[79]
SA Co-N/C (0.05 g/L)	PMS (0.5 mM)	Methoxynaphthalene propionic acid (10 mg/L)	pH (7.0), T (20 °C)	Direct electron transfer, ^1^O_2_, SO_4_^•−^, ^•^OH	electrochemical analysis, EPR, scavenger experiments	100% (15 min)	[81]
SA Co-N-C (0.1 g/L)	PMS (1 mM)	Chloroquine phosphate (10 mg/L)	pH_0_ (6.5), T (25 °C)	Direct electron transfer, ^1^O_2_, SO_4_^•−^, ^•^OH, O_2_^•−^	electrochemical analysis, EPR, scavenger experiments	97.5% (30 min)	[82]
SA-Fe-NC (0.05 g/L)	PMS (2 mM)	Bisphenol A (100 μM)	pH_0_ (6.7), T (30 °C)	^1^O_2_, SO_4_^•−^, ^•^OH, O_2_^•−^	EPR, scavenger experiments	100% (3 min)	[85]
Co-TPML (0.2 g/L)	PMS (2 mM)	Bisphenol A (10 μM)	pH (3.5), T (20 °C)	^•^OH, SO_4_^•−^	EPR, scavenger experiments	90% (5 min)	[86]
Co_SA_-N_4_-C (0.05 g/L)	PDS (2 mM)	Bisphenol A (50 μM)	pH (6.4), T (30 °C)	^•^OH, SO_4_^•−^	EPR, scavenger experiments	100% (4 min)	[87]
Mn-ISAs@CN (0.2 g/L)	PMS (0.2 g/L)	Bisphenol A (20 mg/L)	pH (6), T (25 °C)	^•^OH, SO_4_^•−^, O_2_^•−^, ^1^O_2_	EPR, scavenger experiments	100% (4 min)	[88]
Fe-BNC (0.2 g/L)	PDS (5 mM)	Bisphenol A (20 mg/L)	pH (6.30), T (25 °C)	^1^O_2_, SO_4_^•−^, ^•^OH, O_2_^•−^	EPR, scavenger experiments	100% (20 min)	[89]
SAFe−CN (0.1 g/L)	PMS (0.4 g/L)	O-phenylphenol (10 mg/L)	pH (7), T (25 °C)	Direct electron transfer	electrochemical analysis, EPR, scavenger experiments	100% (30 min)	[90]
SA-Cu/rGO (0.1 g/L)	PMS (0.4 g/L)	Sulfamethoxazole (10 mg/L)	pH (6.0), -	^•^OH, SO_4_^•−^, ^1^O_2_	EPR, scavenger experiments	99.6% (60 min)	[91]
SA-Co CNP (0.2 g/L)	PMS (0.5 g/L)	Acetaminophen (20 mg/L)	pH (7), T (25 °C)	^1^O_2_	EPR, scavenger experiments	92% (10 min)	[92]
Fe-SAC (0.2 g/L)	PMS (0.4 g/L)	Bisphenol A (25 mg/L)	pH (6.5), T (25 °C)	^1^O_2_, ^•^OH	EPR, scavenger experiments	88% (30 min)	[93]
Co-N_3_O_1_ (0.1 g/L)	PMS (1 mM)	Ciprofloxacin (5 mg/L)	pH_0_ (6.5), T (25 °C)	^1^O_2_	EPR, scavenger experiments	100% (20 min)	[94]
Fe-g-C_3_N_4_ (0.1 g/L)	PMS (0.5 mM)	Tetracycline (10 g/L)	pH_0_ (6.5), T (25 °C)	Fe(IV), ^1^O_2_, SO_4_^•−^, ^•^OH, O_2_^•−^	EPR, scavenger experiments	93.3% (40 min)	[95]
ZIF-CoN_3_P-C (0.050 g/L)	PMS (1 mM)	Sulfadiazine (10 mg/L)	pH (3.35), T (20 °C)	^1^O_2_, O_2_^•−^	EPR, scavenger experiments	100% (10 min)	[96]
Fe (MIL)-SAC (0.15 g/L)	PDS (0.4 g/L)	Sulfamethoxazole (20 mg/L)	pH (6.6), T (25 °C)	^1^O_2_	EPR, scavenger experiments	100% (10 min)	[97]
Co SA/CN-900 (0.05 g/L)	PDS (2 mM)	tetracycline hydrochlorid (10 mg/L)	pH_0_ (4.80), T (25 °C)	^1^O_2_, direct electron transfer	electrochemical analysis, EPR, scavenger experiments	100% (6 min)	[98]
Fe−N−C (20 mg/L)	PDS (200 μM)	2,4-dichlorophenol (20 μM)	pH (5.8), T (25 °C)	Fe(V)	electrochemical analysis, EPR, scavenger experiments	100% (90 min)	[99]
N_5_Mn(IV)=O (0.5 g/L)	PMS ( 1.0 mM)	p-chlorophenol (10 mg/L)	pH_0_ (6.7), T (25 °C)	Mn(IV)=O	EPR, scavenger experiments	100% (6 min)	[100]
Cu_SA_-NC (40mg/L)	PDS (0.5 mM)	2,4-dichlorophenol (100 μM)	pH_0_ (6.0), T (25 °C)	Cu(III)	EPR, scavenger experiments	100% (30 min)	[101]
FeSA-NPS@C (20 mg/L)	PMS (0.2 mM)	Ofloxacin (20 μM)	pH (3.5), -	Fe(IV)=O	electrochemical analysis, EPR, scavenger experiments	100% (3 min)	[102]
Fe-SA/PHCNS (0.1 g/L)	PMS (0.2 g/L)	Acetaminophen (10 mg/L)	pH_0_ (7), T (25 °C)	Fe(IV)=O, direct electron transfer, ^1^O_2_, SO_4_^•−^, ^•^OH	EPR, scavenger experiments	100% (10 min)	[103]
ISA-Fe/MC (0.05 g/L)	PMS (0.5 g/L)	Bisphenol A (20 mg/L)	-, T (25 °C)	Direct electron transfer, ^1^O_2_	electrochemical analysis, EPR, scavenger experiments	100% (6 min)	[104]
Co-N_5_/CNT (0.03 g/L)	PMS (0.3 mM)	Sulfamerazine (10 mg/L)	pH_0_ (5.53), T (25 °C)	Direct electron transfer, ^1^O_2_, SO_4_^•−^, ^•^OH, O_2_^•−^	electrochemical analysis, EPR, scavenger experiments	96.3% (30 min)	[105]
BCN/CoN[2 + 2] (0.6 g/L)	PMS (0.6 g/L)	Tetracycline (50 mg/L)	pH (7), T (25 °C)	^1^O_2_	EPR	80% (5 min)	[106]
Cu-SACs (0.2 g/L)	PMS (0.05 mM)	Oxytetracycline (10 mg/L)	-, pH_0_ (6.8)	^1^O_2_, SO_4_^•−^, ^•^OH	EPR, scavenger experiments	96.5% (20 min)	[107]
FeSA-NEPBC (40 mg/L)	PDS (0.4 mM)	Bisphenol S (20 μM)	pH_0_ (6.9), T (25 °C)	Direct electron transfer	electrochemical analysis, EPR, scavenger experiments	100% (40 min)	[108]

* represents catalyst surface complex.

## Data Availability

The data presented in this study are available on request from the corresponding author.
